# COVID-19 and Opioid Use in Appalachian Kentucky: Challenges and Silver Linings

**DOI:** 10.13023/jah.0204.03

**Published:** 2020-09-01

**Authors:** Rachel Vickers-Smith, Hannah L.F. Cooper, April M. Young

**Affiliations:** University of Kentucky; Rollins School of Public Health, Emory University; University of Kentucky

**Keywords:** Appalachia, opioids, COVID-19, rural, drug market, social services

## Abstract

Appalachian Kentucky is currently fighting two public health emergencies – COVID-19 and the opioid epidemic–leaving the area strapped for resources to care for these ongoing crises. During this time, people who use opioids (PWUO) have increased vulnerability to fatal overdoses and drug-related harms (e.g., HIV). Disruption of already limited services posed by COVID-19 could have an especially detrimental impact on the health of PWUO. Though the COVID-19 pandemic is jeopardizing hard-won progress in fighting the opioid epidemic, innovations in state policy and service delivery brought about by the pandemic may improve the health of PWUO long-term if they are retained.

Appalachian Kentucky is currently fighting two public health emergencies—COVID-19 and the decades-long opioid epidemic—leaving the area strapped for resources to care for two ongoing crises. During this time, people who use opioids have increased vulnerability to fatal overdoses, life-threatening withdrawal, relapse, and drug-related harms, such as HIV and hepatitis C (HCV) infection. Indeed, there is significant evidence that the number of opioid overdoses in Kentucky has risen since the start of the pandemic.^1^ Further, access to needed substance-use treatment and harm-reduction services in the region was limited even before COVID-19, and the very existence of many of these services was tenuous due to funding constraints and stigma.^2^,^3^ Disruption of these already strained services posed by COVID-19 and COVID-related public health measures could have an especially detrimental impact on the health of people who use opioids in Appalachian Kentucky.

The COVID pandemic and resulting economic and social impacts has led to deleterious effects on mental health in the general population including increased levels of stress, anxiety, and depression.^4^–^8^ These factors can contribute to the risk of opioid initiation, relapse, and overdose death. Further, due to stay-at-home orders and social distancing practices, individuals may be using opioids in isolation more often—a risk factor for fatal overdose given the limit to bystander’s ability to administer naloxone and/or call emergency services.^1^,^9^

As the novel coronavirus has surged across the globe, it has had a ripple effect on the international illicit drug supply chain, from production to destination markets. This disruption can increase the vulnerability of people who use opioids to drug-related harms such as elevated risk of fatal overdose and HIV/HCV. For example, closed borders and restricted travel have interrupted the heroin supply from Mexico to the U.S.^10^ There are worldwide reports of drug shortages, reduction in purity, and increased prices.^10^ Further, widespread COVID-19–related unemployment has caused economic instability potentially impacting risk of relapse due to associated stress, risk of withdrawal related to affordability of drugs, and increased risk behavior related to drug-seeking (i.e., transactional sex for money and drugs). Alternatively, limited access may actually drive people who use opioids to seek treatment.

In Kentucky, where 20 of the state’s 54 Appalachian counties lacked a syringe service program as of September 2020,^11^ the impact of COVID-19 on program scale-up and utilization remains largely unknown. Preliminary reports from regional health department directors indicate that utilization has remained steady or has increased, but programs have needed to adapt to COVID-19 by moving services outside and shortening the duration of staff–client interactions.

The consequences of more rapid staff–client interactions are currently unclear; the changes could have heterogeneous impacts across communities and among clients. On the one hand, the more rapid, lower threshold access to the program may improve utilization. On the other hand, the brief encounter may prevent staff from talking with clients about HIV/HCV risk reduction, safe injection practices, substance-use treatment, and other client needs.

Although the negative effects of the COVID-19 pandemic are vast, loosened regulations and policies enacted primarily to reduce the community transmission of COVID-19, may have actually improved access for people who use opioids to certain services like telehealth for medication for opioid-use disorder^12^ and unemployment benefits.^13^ In March 2020, SAMHSA (Substance Abuse and Mental Health Services Administration) relaxed the in-person examination requirement for buprenorphine initiation, allowing authorized practitioners to utilize telehealth.^14^ At the beginning of the pandemic, Kentucky’s governor authorized early releases from jail for nearly 700 inmates held for nonviolent offenses,^15^ a positive move to protect incarcerated individuals, although it also confers an overdose risk particularly if services are not available to assist these individuals in community re-entry.

Overall, the COVID-19 pandemic is jeopardizing hard-won progress in fighting the opioid epidemic in Appalachian Kentucky. Although the prevalence of COVID-19 has been relatively low in the area, a surge in infections could be disastrous for people who use opioids and the services on which they rely. These individuals are at a heightened risk for severe illness if they contract COVID-19 because of the immunosuppressive properties of opioids.^16^ Those who sustain COVID-related sequelae, particularly compromised lung function, are at increased risk of fatal overdose due to respiratory depression.^9^ However, innovations in state policy and service delivery brought about by COVID-19 (e.g., low-threshold access to harm reduction, telehealth for opioid-use disorder treatment, decarceration) may improve the health of people who use opioids long-term if they are retained beyond the pandemic.
